# Patterns, Predictors, and Correlates of Problematic Alcohol Use and Remission From Adolescence to Early Midlife

**DOI:** 10.1111/acer.70365

**Published:** 2026-06-30

**Authors:** Megan E. Cooke, Erin Lumpe, Mallory Stephenson, Sally I‐Chun Kuo, Peter B. Barr, Sarah J. Brislin, Holly Poore, Fazil Aliev, Maarit Piirtola, Antti Latvala, Jill A. Rabinowitz, Richard J. Rose, Jaakko Kaprio, Danielle M. Dick, Jessica E. Salvatore

**Affiliations:** ^1^ Department of Psychiatry Robert Wood Johnson Medical School, Rutgers University Piscataway New Jersey USA; ^2^ Rutgers Addiction Research Center Brain Health Institute, Rutgers Health Piscataway New Jersey USA; ^3^ Department of Psychology Rutgers University Piscataway New Jersey USA; ^4^ Virginia Institute for Psychiatric and Behavioral Genetics Virginia Commonwealth University Richmond Virginia USA; ^5^ Department of Cellular, Molecular, and Genetic Medicine Virginia Commonwealth University Richmond Virginia USA; ^6^ Department of Psychiatry & Behavioral Sciences SUNY Downstate Health Sciences University, Brooklyn New York New York USA; ^7^ Institute for Molecular Medicine Finland FIMM University of Helsinki Helsinki Finland; ^8^ Laurea University of Applied Science Vantaa Finland; ^9^ Department of Public Health University of Helsinki Helsinki Finland; ^10^ Institute of Criminology and Legal Policy University of Helsinki Helsinki Finland; ^11^ Department of Psychological and Brain Sciences Indiana University Bloomington Indiana USA

**Keywords:** early midlife, environmental risk, *late‐onset*, *persistent*, polygenic score, problem alcohol use, *remitted*

## Abstract

**Background:**

The goal of this paper is to identify environmental, behavioral, and genetic predictors and early midlife correlates of problematic alcohol Use (PAU) patterns from adolescence to early midlife.

**Methods:**

The data are from 1463 twin individuals (60.5% female) in the population‐based FinnTwin12 cohort born 1983–1987. PAU was assessed at ages 14, 22 and 37 using symptoms of alcohol use disorder (AUD) obtained from psychiatric interviews, mmMAST scores, and the AUDIT. Self‐reported health and well‐being were assessed at age 37. A behavioral and environmental risk index (BERI) was created by summing six risk factors: low socioeconomic status in childhood, family history of AUD, childhood externalizing and internalizing behaviors, adolescent substance use, peer substance use, and stressful life events in adolescence. The twins' genetic predispositions were indexed using polygenic scores for externalizing behavior (PGS_EXT_ ), PAU (PGS_PAU_ ), and depression (PGS_MDD_ ).

**Results:**

Twins were classified into four PAU groups: *Never* (*n* = 599, reference), *Remitted* (*n* = 480), *Persistent* (*n* = 271), and *Late‐onset* (*n* = 113). When included in the same model, both BERI and PGS_EXT_ were significantly associated with *Remitted* PAU, while BERI scores and PGS_PAU_ were significantly associated with *Persistent* PAU. Compared to the *Never* PAU group, individuals in the *Persistent* and *Late‐onset* PAU groups reported lower educational attainment, poorer physical health, lower relationship satisfaction, lower life satisfaction, a worse financial situation, more depressive symptoms, recurrent pain, and sleep problems, and were less likely to be partnered or have children.

**Conclusions:**

*Persistent* PAU was associated with the most negative outcomes in early midlife, and childhood and adolescent risk factors and genetic risk as indexed by PGS_PAU_ were strong predictors of group membership. *Late‐onset* PAU may be the result of novel stressors in adulthood, given the lack of association with childhood and adolescent environmental risk or genetic risk.

## Graphical abstract

*Late‐onset* problem alcohol use (PAU) may be the result of novel stressors in adulthood, given the lack of association with childhood and adolescent environmental risk or genetic risk. Encouragingly, *Remitted* PAU resembled *Never* PAU in early midlife, despite having elevated genetic and prior environmental risk.

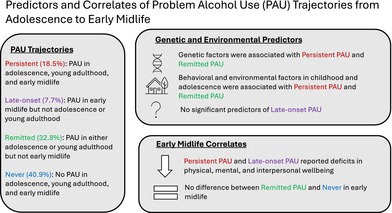

## 1 Introduction

Alcohol use was the leading risk factor for disability‐adjusted life years globally for persons aged 25 to 49 years in 2019 (Murray et al. 2020). Studies of the natural history of problem alcohol use (PAU), including alcohol use disorder (AUD), underscore its heterogeneity in terms of clinical features such as age of onset and whether one has a persistent versus remitting course (Clark 2004; Vaillant 1995; Verges et al. 2012). Yet, with few exceptions (McCutcheon et al. 2014; Meier et al. 2013), the field's understanding of what prospectively predicts the course of PAU, or the associations between PAU course and health and psychosocial functioning in early midlife remains understudied. This gap exists despite the fact that PAU remains common in early midlife (Capaldi et al. 2015; Schulenberg et al. 2016). Furthermore, much of what is known about predictors and correlates of PAU course has been inferred from cross‐sectional retrospective reports, or in highly selected or non‐representative samples (Babor 1996; Clark 2004; Jellinek 1960; Vaillant 1995). For this reason, longitudinal population‐based samples with detailed clinical information are especially valuable for answering questions about the correlates and consequences of problem alcohol use. In this study, we aimed to extend the knowledge of the course of alcohol misuse into the early midlife developmental period by examining whether patterns of problematic alcohol use across adolescence through early midlife are related to adult psychosocial functioning in a population‐based sample. We further tested hypotheses about behavioral, environmental, and genetic risk factors that may be prospectively associated with the course of PAU.

There is considerable heterogeneity in the development and course of PAU (Tucker et al. 2020). Prospective studies have consistently identified several distinct subtypes of alcohol use (Jackson and Sartor 2016; Sher et al. 2011), most commonly a low or no use or problems group, a high or consistent use or problems group, a group whose use or problems has decreased over time, and a group whose use or problems have increased over time. However, most of these studies have classified subtypes based on frequency and quantity of use, with a few exceptions incorporating diagnostic clinical interview data (e.g., Meier et al. 2013; Seeley et al. 2019). Yet, prospective, longitudinal data capturing both consumption patterns and symptom data are especially valuable (Jackson et al. 2014) for better understanding the dynamic nature of PAU across development, including *Persistent*, *Remitting*, and *Late‐onset* trajectories.

The development of PAU is the result of the interplay between genetic and environmental influences (Verhulst et al. 2015) with the relevant importance of these influences changing over time (Kendler et al. 2008). Polygenic scores (PGS), which capture genetic variation across the genome, have shown consistent associations with PAU (Kuo et al. 2021; Nurnberger et al. 2022; Salvatore et al. 2014), indicating unique and shared genetic vulnerability with other psychiatric disorders and traits. Risk factors for PAU span many domains including familial (i.e., childhood SES, family history; Barr et al. 2018), mental health comorbidities (i.e., childhood conduct disorder and childhood depression; Fergusson et al. 2007; Hussong et al. 2011), early substance use (Odgers et al. 2008), and stressful life events (Hoyland and Latendresse 2018; Sheerin et al. 2021). Previous research has used cumulative risk indices to identify individuals at risk for developing substance use disorders (Barr et al. 2022; Meier et al. 2016).

Differentiating among developmental courses of PAU from adolescence to early midlife is crucial, as PAU in early midlife is common (Capaldi et al. 2015; Schulenberg et al. 2016) and robustly associated with poorer functioning in many demographic (i.e., family formation), physical health (i.e., self‐rated health, recurrent pain, physical fitness, and sleep difficulties), and well‐being (i.e., life satisfaction and psychological health) domains (Lumpe et al. 2025; Schulenberg et al. 2016). Early midlife represents a unique developmental stage with regard to PAU. Many physical and social consequences from long‐term alcohol use and misuse may begin to emerge (Lachman 2015; Topiwala et al. 2017), making it a pivotal time to seek treatment (Blanco et al. 2015; Grant et al. 2015), and yet new onsets of PAU also emerge at this stage (Verges et al. 2012).

Finally, there is consistent evidence for sex differences in alcohol outcomes, with some evidence that the magnitude and direction of effect changes across developmental stages (Nolen‐Hoeksema 2004; Nolen‐Hoeksema and Hilt 2006; Windle 2016). Recently observed increases in females' PAU and decreases in males' PAU have resulted in a decline in the magnitude of sex differences in PAU (Keyes et al. 2011; White et al. 2015). These broad societal shifts in sex differences in PAU underscore the importance of examining sex differences in predictors and correlates of PAU across the lifespan.

The goal of the current study was to identify the genetic, behavioral, and environmental predictors of and sociodemographic, interpersonal, and health correlates of *Persistent*, *Late‐onset*, and *Remitted* PAU in early midlife. Using data from the FinnTwin12 cohort study (Cooke et al. 2025), an ongoing longitudinal study of Finnish twins recruited at age 12 and most recently followed up in early midlife, we sought to quantify the prevalence of *Persistent*, *Late‐onset*, and *Remitted* courses of PAU between adolescence and early midlife, identify early midlife correlates of these groups, and quantify the association between group membership and behavioral, environmental, and genetic risk factors measured across development. We also tested for potential sex differences in the above analyses.

## 2 Materials and Methods

### 2.1 Sample

Participants were from the FinnTwin12 cohort, a population‐based study of Finnish twins born between 1983 and 1987 and originally identified through Finland's Central Population Registry. Key information regarding the study design can be found in Cooke et al. (2025). The full epidemiological sample included 5184 twins from 2705 families initially recruited via mailers in the year that twins reached age 12. All twins were invited to participate in follow‐ups at age 14 (92% retention), age 22 (66% retention), and age 35 (40% retention; Cooke et al. 2025). A subsample of intensively studied twins (*n* = 1854) enriched for parental alcohol misuse participated in laboratory‐based assessments and psychiatric interviews at ages 14 and 22 (73% retention). In addition, 1260 intensively studied twins (526 monozygotic [MZ] twins) provided a DNA sample at age 22. The final analytic sample (*n* = 1463; 529 MZ twins, 154 complete MZ twin pairs, 849 dizygotic [DZ] twins, 93 complete same sex DZ pairs, 90 complete opposite sex DZ pairs) included twins with relevant data at 14 and 22 and who completed the self‐report questionnaires at age 37.

### 2.2 Measures

#### 2.2.1 Problem Alcohol Use (PAU)

How PAU was operationalized at each timepoint is described below and summarized in Table S1 found in the Supporting Information.

##### 2.2.1.1 PAU in Adolescence

Twins in the intensively studied sample who had a diagnosis of alcohol abuse or dependence from the Finnish adaptation of the Semi‐Structured Assessment for the Genetics of Alcoholism (SSAGA; Sihvola et al. 2008) at age 14 were classified as having PAU in adolescence. Twins in the intensively studied sample who did not have a diagnosis according to the SSAGA and twins from the epidemiological sample, who were not administered the SSAGA and who reported drinking less than once per month at the age 14 questionnaire, were classified as not having PAU.

##### 2.2.1.2 PAU in Young Adulthood

Twins in the intensive sample who had a diagnosis of alcohol abuse or dependence from the SSAGA (Sihvola et al. 2008) at age 22 were classified as having PAU in young adulthood. For twins in the epidemiological sample who were not administered the SSAGA in young adulthood, we used scores on the Malmö modification of the brief Michigan Alcoholism Screening Test (mmMAST, Kristenson and Trell 1982) at age 22 to determine PAU status. Twins who endorsed 4 or more items out of 11 on the mmMAST were classified as having PAU in young adulthood based on prior research in Finnish twins using this cutoff (Sipilä et al. 2023).

##### 2.2.1.3 PAU in Early Midlife

Twins completed the 10‐item Alcohol Use Disorders Identification Test (AUDIT); (Cooke et al. 2025; Saunders et al. 1993) at age 37, which includes seven items measuring problems from alcohol use. Of the seven problem alcohol use items, five assess frequency of alcohol problems specifically within the past year, such as a loss of control over drinking, not meeting expectations due to drinking, drinking first thing in the morning, feelings of guilt or remorse after drinking, and an inability to remember what happened the night before. Response options were (0) “never,” (1) “less than monthly,” (2) “monthly,” (3) “weekly,” or (4) “daily or almost daily.” The five items were summed to create a score assessing past‐year PAU, with scores of 2 or greater classified as PAU in early midlife. This threshold was chosen to mirror the diagnostic criteria from the SSAGA, which requires that at least two symptoms occur at least three times in a 12‐month period. Rates of PAU in early midlife did not significantly differ between the epidemiological sample and the intensively studied sample (*X* ^2^ = 3.26, *p* = 0.071).

##### 2.2.1.4 PAU Trajectory Groups

PAU in adolescence, young adulthood, and early midlife (defined above) were used to create the PAU trajectory groups. Twins were classified as not having lifetime alcohol problems (characterized as the “*Never*” group) if they met the following criteria: (1) no PAU in adolescence, young adulthood, and early midlife, (2) and responded “No” to each of the final AUDIT questions (i.e., “have you or someone you know been injured because of your drinking?” and “has a relative, friend, doctor, or other health care worker been concerned about your drinking or suggested that you cut down?”). Twins were classified into the “*Persistent*” group if they had PAU in adolescence or young adulthood or both, and PAU in early midlife. Twins were classified into the “*Late‐onset*” group if they had (1) no PAU in adolescence and young adulthood and (2) did have PAU in early midlife. Lastly, twins were classified into the “*Remitted*” group if they (1) had PAU in either adolescence or young adulthood or both, (2) did not have PAU in early midlife, and (3) did not endorse past‐year alcohol problems on the final two AUDIT questions.

#### 2.2.2 Early Midlife Correlates

Detailed information on the early midlife correlates with references can be found in the Data S1. A summary of each measure is provided below.

##### 2.2.2.1 Socioeconomic Status Indicators

Twins reported their highest level of education achieved. Responses were recoded to compulsory education only, vocational secondary education, academic secondary school, and tertiary education. Twins reported on their financial situation using an ordinal scale with five response options from “very good” to “very poor.” Response options were coded such that higher scores represented a better financial situation.

##### 2.2.2.2 Relationship Indicators

Twins who reported living with a spouse or partner or reported currently being in a relationship were coded as partnered. Twins who reported being in a relationship were administered three subscales (relationship consensus, relationship satisfaction, and relationship cohesion) from the Revised Dyadic Adjustment Scale (RDAS). Relationship consensus (*α* = 0.69) measured the ability to achieve consensus in decision‐making in the relationship. Relationship satisfaction (*α* = 0.79) measured the extent to which they were satisfied in the relationship. Relationship cohesion (*α* = 0.73) measured the degree to which partners participate in activities together. All items were recoded (if necessary) such that higher scores represented more positive relationship outcomes. Items were then summed within each subscale. Twins reported whether they were a biological father/mother and whether there were non‐biological children living in their household. Parenthood status was coded as a binary variable indicating whether the twin had biological children (regardless of residential status) or non‐biological children living in the same household.

##### 2.2.2.3 Health‐Related Indicators

Twins reported on their current health and current physical fitness using an ordinal scale with five response options from “very good” to “very poor” for each outcome. Response options were coded such that higher scores represented better self‐reported health or physical fitness. Twins reported the frequency of experiencing “headaches,” “low back pain,” “neck or shoulder pain,” “difficulty getting to sleep,” and “waking up during sleep” during the past 6 months using an ordinal scale with five response options from “less often or never” to “almost daily.” An endorsement of weekly or more frequent pain as “headaches,” “low back pain,” or “neck or shoulder pain” was coded as recurrent pain. An endorsement of weekly or more frequent difficulty getting to sleep or waking up during sleep was coded as recurrent sleep difficulties.

##### 2.2.2.4 Mental Health Related Indicators

Twins were administered the Satisfaction with Life Scale, a 5‐item scale used to measure global cognitive judgments of one's life satisfaction. Scores were summed to create a scale from 5 to 35, with a score of 20 being neutral (*α* = 0.89). Twins were administered the modified 8‐item Center for Epidemiological Studies Depression Scale (CES‐D), a self‐report questionnaire designed to measure frequency of past‐week depressive symptoms. Scores (0 to 3) were summed to create a range between 0 and 24, with higher scores indicating more severe and frequent depressive symptoms (*α* = 0.8).

#### 2.2.3 Behavioral, Environmental, and Genetic Risk Index

Informed by Barr et al. (2022), we created a behavioral and environmental risk index (BERI) to represent aggregate risk for AUD using a sum score of previously established risk factors. BERI risk factors include dichotomized variables representing childhood socioeconomic status, family history of alcohol use disorder, childhood externalizing behavior, childhood internalizing symptoms, early initiation of substance use, adolescent alcohol use, adolescent tobacco use, adolescent cannabis use, peer substance use, and stressful life events. Descriptions of each of the variables in the index and their coding can be found in Table 1 with further details available in the Supporting Information.

**TABLE 1:** Items included in the behavioral and environmental risk index (BERI).

Low socioeconomic status	Parents with less than the compulsory level of education or a manual, low‐wage, or low‐skill occupation
Family history of AUD	AUD diagnosis in parents of twins
Childhood externalizing behavior	Diagnosis of conduct disorder or oppositional defiance disorder at age 14 or earlier
Childhood internalizing behavior	Diagnosis of major depression at age 14 or earlier
Substance use initiation	Any substance use at age 14 or earlier
Adolescent alcohol use	5 or more days/week of alcohol use at age 17 or earlier
Adolescent tobacco use	Daily tobacco use at age 17 or earlier
Adolescent cannabis use	5 or more days/week of cannabis use at age 17 or earlier
Peer substance use	Most friends smoke, drink, and/or have tried drugs
Stressful life events	4 or more stressful life events

*Note:* An extended description of BERI items is available in the supplement (Table S2).

#### 2.2.4 Polygenic Score Creation (PGS)

Informed by significant associations between AUD and PGS representing externalizing behavior and alcohol problems (Barr et al. 2022), summary statistics from a GWAS of externalizing behavior (Karlsson Linnér et al. 2021) and alcohol‐specific liability (Poore et al. 2026) were used to create polygenic scores representing genetic risk for externalizing behavior and alcohol specific genetic effects, respectively. We note that the PGS for alcohol‐specific effects accounts for genetic variation shared between alcohol use and general externalizing behavior and therefore represents genetic liability specific to problem alcohol use. Given comorbidity between AUD and depression, we also created a PGS representing genetic risk for depression using summary statistics from a GWAS of major depression (Adams et al. 2025). All PGS were created using PRS‐CS (Ge et al. 2019). We then regressed each PGS on the first 10 ancestral principal components. Each PGS was standardized prior to their inclusion in subsequent analyses. Additional information on the genotyping and quality control steps can be found in the Data S1.

### 2.3 Analytic Plan

The following analytic plan was preregistered (osf.io/ubcqn). To examine the associations between the behavioral, environmental, and genetic risk factors and the PAU trajectory groups, we conducted a series of multinomial logistic regression models predicting the outcome variable of PAU group membership described above. We conducted the following models using the *mclogit* package in R: (1) a baseline model (age and sex), (2) a genetic risk model for each PGS (baseline + PGS), (3) a BERI model (baseline + BERI), and (4) combined model (baseline + PGS + BERI). These models also accounted for family structure in the FT12 dataset by including family ID as a random effect in all models. We examined sex differences in the combined model by adding a sex by BERI and sex by PGS interaction term.

To examine early midlife correlates of each trajectory group, we used linear mixed models and mixed effects logistic regression models, which account for the family structure in the data through including family ID as a random effect, and set up the following comparisons: (1) a model comparing each PAU trajectory group to the *Never* group (i.e., *Persistent* vs. *Never*, *Late onset* vs. *Never*, *Remitted* vs. *Never*), and (2) a model including only the PAU trajectory groups comparing *Remitted* or *Late‐onset* groups to the *Persistent* group. We used the *lme4* and *ordinal* packages in R to run the above models depending on the coding of the outcome. Sex was included as a covariate in all models. Finally, to examine sex differences, we added a sex by group interaction term to the models. To account for multiple testing in the early midlife correlate analyses, we used a Bonferroni correction to adjust the *p*‐value threshold for significance to 0.003 based on the 15 early midlife correlates identified in the preregistration.

## 3 Results

### 3.1 Sample Representativeness

Compared to the full epidemiological sample collected at age 12, twins in the analytic sample for the current study were more likely to be female (OR: 2.31, 95% CI: 1.90–2.83) and monozygotic twins (OR: 1.44, 95% CI: 1.15–1.80). Twins in the analytic sample had lower levels of aggression (OR: 0.80, 95% CI: 0.65–0.99), and impulsivity or hyperactivity (0.65, 95% CI: 0.55–0.77) as reported by the parents at age 12. Parents' reports of anxiety and depression at age 12 were not significantly associated with inclusion in the analytic sample. Twins' reports of their frequency of alcohol use or frequency of intoxication at age 14 were not significantly associated with inclusion in the analytic sample.

### 3.2 Descriptives

Descriptive statistics for the analytic cohort, stratified by PAU group, are summarized in Table 2. Out of the total sample, 1.6% had PAU in adolescence, 50.7% had PAU in young adulthood, and 26.2% had PAU in early midlife. Descriptive statistics stratified by sex can be found in Table S3. *Never* PAU was the largest single group (40.9%), but most twins, across both sexes, experienced PAU and could be classified into one of the other three groups (59%). Of the 356 complete pairs of twins in the analytic sample, 172 pairs (48.3%) were concordant for PAU trajectory. The zygosity breakdown of the concordant pairs was 88 (51.2%) monozygotic twin pairs, 40 (23.3%) dizygotic same sex twin pairs, and 33 (19.2%) dizygotic opposite sex twin pairs. The PAU group breakdown of the concordant pairs was 96 *Never* PAU pairs (55.8%), 44 *Remitted* PAU pairs (25.6%), 26 *Persistent* PAU pairs (15.1%), and 6 *Late‐onset* PAU pairs (3.5%). On average, the twins endorsed 2.4 (SD = 1.4) indicators in the BERI. The correlation between twins on the BERI index was 0.76 in the overall sample (95% CI: 0.72–0.79), 0.85 (95% CI: 0.81–0.88) among MZ twins, 0.75 (95% CI: 0.67–0.81) among same sex DZ twins, and 0.62 (95% CI: 0.50–0.71) among opposite sex DZ twins. Below we present the results from our primary analyses starting with associations examining the genetic and environmental predictors of group membership and subsequently associations between group membership and early midlife indicators of health and wellbeing.

**TABLE 2:** Analytic sample descriptives.

	*Never*	*Persistent*	*Late‐onset*	*Remitted*
	*n* (%)^a^	*n* (%)^a^	*n* (%)^a^	*n* (%)^a^
*N*	599 (40.9)	271 (18.5)	113 (7.7)	480 (32.8)
Zygosity				
MZ	255 (42.6)	91 (33.6)	32 (28.3)	151 (31.5)
DZ SS	158 (26.4)	76 (28.0)	41 (36.3)	132 (27.5)
DZ OS	157 (26.2)	88 (32.5)	35 (31.0)	162 (33.8)
Unknown zygosity	28 (4.7)	11 (4.1)	4 (3.5)	26 (5.4)
BERI	2.1 (1.3)	2.8 (1.4)	2.7 (1.4)	2.5 (1.4)
Education				
Compulsory education only	3 (0.5)	7 (2.6)	3 (2.7)	9 (1.9)
Vocational secondary	116 (19.4)	82 (30.3)	25 (22.1)	92 (19.2)
Academic secondary	57 (9.5)	33 (12.2)	10 (8.8)	55 (11.5)
Tertiary education	422 (70.5)	148 (54.6)	75 (66.4)	324 (67.5)
Financial situation				
Very good	130 (21.7)	31 (11.4)	17 (15.0)	77 (16.0)
Fairly good	239 (39.9)	113 (41.7)	53 (39.8)	208 (43.3)
Moderate	191 (31.9)	101 (37.3)	35 (31.0)	164 (34.2)
Fairly poor	34 (5.7)	24 (8.9)	7 (6.2)	22 (4.6)
Very poor	4 (0.7)	2 (0.7)	1 (0.9)	9 (1.9)
Partnered	470 (78.5)	167 (61.6)	73 (64.6)	357 (74.4)
Relationship dynamics, mean (SD)				
Consensus	23.2 (3.2)	22.1 (3.1)	22.3 (3.2)	22.7 (3.1)
Satisfaction	14.8 (3.1)	14.3 (3.2)	13.7 (3.1)	14.6 (3.2)
Cohesion	12.1 (3.0)	11.9 (2.9)	11.9 (3.0)	12.0 (3.0)
Parenthood	408 (68.1)	132 (48.7)	57 (50.4)	323 (67.3)
Self‐rated health				
Very good	112 (18.7)	24 (8.9)	16 (14.2)	75 (15.6)
Quite good	340 (56.8)	150 (55.4)	49 (43.4)	258 (53.8)
Average	130 (21.7)	79 (29.2)	44 (38.9)	126 (26.3)
Quite poor	16 (2.7)	18 (6.6)	4 (3.5)	18 (3.8)
Very poor	1 (0.2)	0 (0.0)	0 (0.0)	2 (0.4)
Self‐rated physical fitness				
Very good	66 (11.0)	20 (7.4)	10 (8.8)	34 (7.1)
Quite good	283 (47.2)	125 (46.1)	47 (41.6)	237 (49.4)
Satisfactory	197 (32.9)	89 (32.8)	39 (34.5)	164 (34.2)
Quite poor	47 (7.8)	36 (13.3)	17 (15.0)	39 (8.1)
Very poor	5 (0.8)	1 (0.4)	0 (0.0)	6 (1.3)
Recurrent pain	302 (50.4)	136 (50.2)	73 (64.6)	265 (55.2)
Recurrent sleep difficulties	224 (37.4)	128 (47.2)	51 (45.1)	197 (41.0)
Life satisfaction, mean (SD)	25.8 (6.0)	23.4 (6.3)	23.5 (6.3)	24.9 (6.0)
Depressive symptoms, mean (SD)	4.4 (3.4)	6.0 (3.9)	5.7 (3.5)	4.9 (3.7)

Abbreviations: DZ OS, opposite sex dizygotic twins; DZ SS, same sex dizygotic twins; MZ, monozygotic twins; PAU, problem alcohol use.

^a^ *n* (%) if not otherwise stated.

### 3.3 BERI and PGS Predictors of PAU Groups

Table 3 shows the results of the multivariable model examining the BERI and PGS predicting PAU group membership. Results from the univariable models can be found in Table S4. PGS_MDD_ was not associated with any of the PAU groups in the multivariable model. PGS_EXT_ and PGS_ALC‐sp_ were uniquely associated with *Remitted* and *Persistent* PAU, respectively, in the multivariable model. The BERI was significantly associated with both *Remitted* and *Persistent* PAU. Sex did not significantly moderate any of the BERI or PGS associations in the combined model.

**TABLE 3:** Behavioral and genetics predictors of PAU groups compared to *Never* group.

	*Remitted*			*Persistent*			*Late onset*		
	OR	CI	*p*	OR	CI	*p*	OR	CI	*p*
BERI	**1.36**	**1.16–1.60**	**0.0001**	**1.42**	**1.18–1.71**	**0.0003**	1.17	0.94–1.46	0.165
PGS_EXT_	**1.49**	**1.18–1.90**	**0.001**	1.29	0.97–1.70	0.081	1.10	0.79–1.52	0.580
PGS_MDD_	0.92	0.73–1.16	0.460	1.27	0.96–1.68	0.093	1.01	0.74–1.39	0.949
PGS_ALC‐sp_	1.25	0.99–1.57	0.056	**1.48**	**1.13–1.93**	**0.004**	0.97	0.71–1.31	0.836

*Note:* The *Never* group was the reference group for all comparisons. The ORs for any PGS predictors represent a 1 standard deviation increase in the PGS. The ORs for the BERI represent an increase of one risk factor on the BERI. Bolded values represent significant associations after correcting for multiple testing.

Abbreviations: ALC‐sp, alcohol‐specific effects; BERI, behavioral and environmental risk index; CI, 95% confidence intervals; EXT, externalizing behavior; MDD, major depressive disorder; OR, odds ratio; PGS = polygenic score.

### 3.4 Early Midlife Correlates of PAU Groups

**FIGURE 1:**
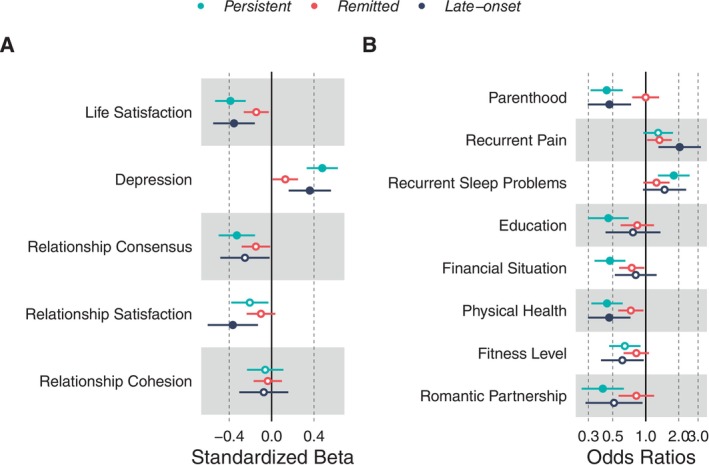
Forest plot of early midlife correlates of PAU group with *Never* as the reference group. The reference category for all analyses in the figure is the *Never* group. Filled in circles represent statistically significant associations adjusted for multiple tests.

Figure 1 shows the associations between PAU group membership, with *Never* PAU as the reference category for all groups, and the early midlife correlates of interest. Exact estimates and confidence intervals are shown in Table 4. *Persistent* PAU was associated with poorer financial situation, physical health, lower educational attainment, life satisfaction, relationship consensus, and higher depression scores. Individuals in the *Persistent* PAU group were also more likely to have recurrent sleep problems and less likely to be partnered or be parents when compared to individuals in the *Never* group. *Late‐onset* PAU was associated with worse physical health, lower life and relationship satisfaction, and higher depression scores. Individuals in the *Late‐onset* PAU group were less likely to be parents and more likely to have recurrent pain. *Remitted* PAU was not significantly associated with any early midlife correlates compared to the *Never* group. Sex did not significantly moderate any of the associations between any early midlife correlates and *Persistent* PAU, *Late‐onset* PAU, or *Remitted* PAU.

**TABLE 4:** Estimates and 95% confidence intervals for early midlife correlates with *Never* PAU as the reference.

	*Remitted*			*Persistent*			*Late‐onset*		
	OR	CI	*p*	OR	CI	*p*	OR	CI	*p*
Education	0.84	0.59, 1.19	0.32	**0.46**	**0.30, 0.70**	**< 0.001**	0.77	0.43, 1.36	0.36
Financial situation	0.74	0.57, 0.97	0.03	**0.47**	**0.34, 0.65**	**< 0.001**	0.81	0.52, 1.19	0.35
Partner status	0.82	0.57, 1.19	0.30	**0.41**	**0.26, 0.63**	**< 0.001**	0.51	0.28, 0.93	0.03
Parenthood status	0.998	0.75, 1.32	0.99	**0.44**	**0.31, 0.62**	**< 0.001**	**0.47**	**0.30, 0.73**	0.**001**
Self‐rated health	0.73	0.56, 0.95	0.02	**0.44**	**0.32, 0.62**	**< 0.001**	**0.46**	**0.30, 0.73**	**< 0.001**
Physical fitness	0.82	0.63, 1.07	0.14	0.64	0.46, 0.89	0.008	0.61	0.39, 0.96	0.03
Recurrent pain	1.33	1.03, 1.73	0.03	1.30	0.95, 1.77	0.10	**2.04**	**1.31, 3.19**	0.**002**
Recurrent sleep issues	1.25	0.95, 1.65	0.11	**1.80**	**1.29, 2.51**	0.**001**	1.49	0.94, 2.33	0.09
	***β***	**CI**		***β***	**CI**		***β***	**CI**	
Relationship consensus	−0.15	−0.28, −0.01	0.03	**−0.33**	**−0.50, −0.16**	**< 0.001**	−0.25	−0.48, −0.02	0.03
Relationship satisfaction	−0.10	−0.24, 0.04	0.15	−0.21	−0.38, −0.03	0.02	**−0.37**	**−0.60, −0.13**	0.**003**
Relationship cohesion	−0.04	−0.17, 0.10	0.60	−0.06	−0.23, 0.11	0.49	−0.07	−0.31, 0.16	0.53
Depressive symptoms	0.13	0.01, 0.25	0.03	**0.48**	**0.33, 0.63**	**< 0.001**	**0.36**	**0.16, 0.56**	**< 0.001**
Life satisfaction	−0.15	−0.26, −0.03	0.02	**−0.39**	**−0.53, −0.25**	**< 0.001**	**−0.36**	**−0.55, −0.16**	**< 0.001**

*Note:* The reference category for all analyses in the table is the *Never* group. Bolded values represent significant associations after correcting for multiple testing.

Abbreviations: CI, 95% confidence intervals; OR, odds ratio.

**FIGURE 2:**
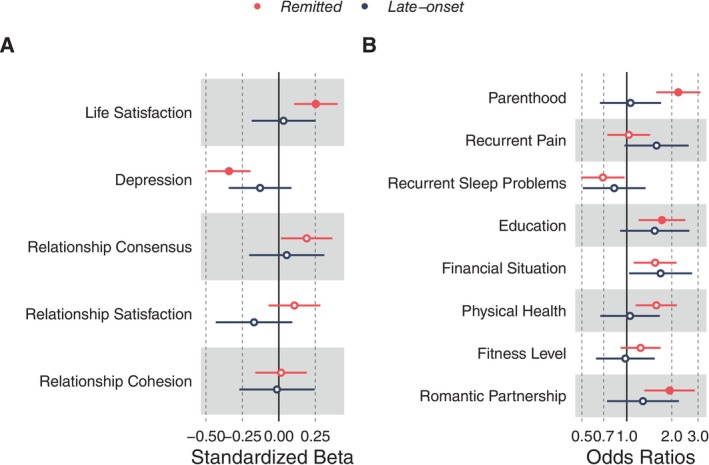
Forest plot of early midlife correlates with *Persistent* as the reference group. The reference category for all analyses in the figure is the *Persistent* group. Filled in circles represent statistically significant associations adjusted for multiple tests.

Figure 2 shows the associations between PAU group membership, with the *Persistent* group as the reference category for comparisons with *Late‐onset* or *Remitted* PAU, and the same series of early midlife correlates above. Exact estimates and confidence intervals are shown in Table 5. After adjusting for multiple comparisons, *Remitted* PAU was associated with higher educational attainment, higher life satisfaction, fewer depressive symptoms, and greater odds of being partnered or being a parent. There were no other significant associations between early midlife correlates and *Late‐onset* or *Remitted* PAU with *Persistent* PAU as the reference group. Sex did not significantly moderate the associations between any of the early midlife correlates and *Late‐onset* or *Remitted* PAU.

**TABLE 5:** Estimates and 95% confidence intervals for early midlife correlates with *Persistent* as a reference group.

	*Remitted*			*Late‐onset*		
	OR	CI	*p*	OR	CI	*p*
Education	**1.72**	**1.20, 2.47**	0.**003**	1.54	0.90, 2.62	0.11
Financial situation	1.55	1.11, 2.15	0.009	1.68	1.04, 2.74	0.04
Partner status	**1.94**	**1.31, 2.86**	0.**001**	1.28	0.74, 2.23	0.38
Parenthood status	**2.21**	**1.58, 3.11**	**< 0.001**	1.06	0.66, 1.70	0.81
Self‐rated health	1.58	1.15, 2.17	0.005	1.05	0.66, 1.66	0.83
Physical fitness	1.24	0.91, 1.68	0.18	0.98	0.62, 1.54	0.92
Recurrent pain	1.03	0.74, 1.43	0.86	1.58	0.97, 2.60	0.07
Recurrent sleep issues	0.69	0.50, 0.97	0.03	0.82	0.51, 1.34	0.43
	***β***	**CI**		***β***	**CI**	
Relationship consensus	0.19	0.02, 0.37	0.03	0.05	−0.20, 0.31	0.68
Relationship satisfaction	0.11	−0.07, 0.29	0.24	−0.17	−0.43, 0.09	0.20
Relationship cohesion	0.02	−0.16, 0.19	0.87	−0.01	−0.27, 0.25	0.92
Depressive symptoms	**−0.34**	**−0.49, −0.19**	**< 0.001**	−0.13	−0.34, 0.09	0.24
Life satisfaction	**0.25**	**0.10, 0.40**	**< 0.001**	0.03	−0.19, 0.25	0.77

*Note:* The reference category for all analyses in the table is the *Persistent* group. Bolded values represent significant associations after correcting for multiple testing.

Abbreviations: CI, 95% confidence intervals; OR, odds ratio.

## 4 Discussion

In the current study, we sought to identify behavioral, environmental, and genetic predictors and early midlife correlates of trajectories of PAU from ages 14 through 37. The BERI was a strong predictor of *Persistent* PAU and *Remitted* PAU across univariable and combined models. Genetic risk for general externalizing behavior and specific to problem alcohol use was associated with both *Persistent* PAU and *Remitted* PAU in the univariable models. However, in the combined model, genetic risk for externalizing behavior was only associated with *Remitted* PAU and alcohol‐specific genetic risk was only associated with *Persistent* PAU. In the combined model, neither the BERI nor genetic risk were associated with *Late‐onset* PAU. Compared to the group of individuals who had never experienced PAU, membership in the *Persistent*, *Remitted*, and *Late‐onset* groups was associated with a unique constellation of correlates in early midlife indicating specific harms associated with each trajectory of PAU. We discuss the findings for each pattern of PAU in turn below.

*Persistent* PAU was associated with the highest number of adverse outcomes in early midlife. Specifically, individuals with *Persistent* PAU reported worse financial situation, worse physical health, lower educational attainment, lower life satisfaction, lower relationship consensus, increased depression, and greater sleep problems. Those with *Persistent* PAU were also less likely to be partnered or parents. These findings are in line with previous research demonstrating that PAU which onsets in adolescence and persists into adulthood is associated with greater severity and worse outcomes (Clark et al. 1998; Foster et al. 2014; Hicks et al. 2010). The current study reveals novel associations between *Persistent* PAU and worse physical health, lower life satisfaction, lower relationship consensus, and increased depression to this body of literature. Our findings that genetic risk for both externalizing behavior and alcohol‐specific effects were associated with *Persistent* PAU is in line with research showing genetic liability is more strongly related to early‐onset and more severe cases of PAU (Milne et al. 2009; Nurnberger et al. 2022). Additionally, prior research (Kendler et al. 2011; Meyers et al. 2014) has shown that specific genetic risk factors (e.g., PGS_PAU_) have a stronger influence in adulthood compared to nonspecific genetic risk factors (e.g., PGS_EXT_) which is further supported by the robustness of the association between PGS_Alc‐sp_ and *Persistent* PAU in the combined model.

In contrast to *Persistent* PAU, *Remitted* PAU was not significantly associated with any adverse outcomes in early midlife compared to *Never* PAU. The phenomenon of “maturing out” of problem drinking, whereby heavy drinking and associated problems tend to decline after a peak at age 22, is well documented (Lee and Sher 2018; O'Malley 2004). It is thought that this developmental decrease in alcohol use is driven by “role incompatibility” (Dawson et al. 2006), as the individual enters the workforce, gets married, and has children, as well as changes in personality (Littlefield et al. 2009). Our finding that those with *Remitted* PAU are no less likely to have a romantic partnership or children than individuals who have never had PAU provides additional support for the influence of role transitions on PAU. Interestingly, despite their success and adaptation across these domains, these individuals had elevated genetic and behavioral/environmental risk for problem alcohol use compared to those who never had PAU. Taken together, the results among individuals with *Remitted* PAU provide encouraging insights that it is possible to reduce PAU in early midlife despite genetic and behavioral risk factors in adolescence and young adulthood, and that those with a remitting trajectory are as healthy on the indicators measured in early midlife as those who never engaged in PAU.

Our findings further suggested that *Late‐onset* PAU had features in common with both the *Persistent* and *Remitted* PAU groups with regard to early midlife health and well‐being. Like *Persistent* PAU, *Late‐onset* PAU was associated with lower life satisfaction, higher depression, a lower likelihood of being a parent, and worse physical health compared to *Never* PAU. However, like *Remitted* PAU, there was no difference in relationship consensus or cohesion, sleep problems, education, financial situation, fitness level, and romantic partnership compared to individuals who never had PAU. Most of these correlates replicate prior literature examining PAU in early midlife (Lumpe et al. 2025; Schulenberg et al. 2016), as well as studies focusing on PAU onset in midlife (Khalifeh et al. 2025; Meier et al. 2013). Unlike *Persistent* PAU and *Remitted* PAU, *Late‐onset* PAU was uniquely associated with lower relationship satisfaction compared to individuals who never had PAU. While we are unable to determine the direction of this effect, this association could be because these individuals' PAU began during the relationship and therefore may currently differ from their partner's substance use. The negative effect of discordance in substance use between partners on relationship outcomes, like satisfaction, has been well documented (Homish and Leonard 2007; Leonard et al. 2014; Meiklejohn et al. 2012; Whisman et al. 2006). Interestingly, *Late‐onset* PAU was not associated with genetic risk or behavioral/environmental risk factors accumulated earlier in life in the combined model. This contrasts with previous studies which have found that familial history and prior risk factors are associated with PAU in midlife (Khalifeh et al. 2025; Meier et al. 2013; Stephenson et al. 2025; Warner et al. 2007). However, many of these studies did not distinguish midlife onset of PAU from a persistent course. Our findings indicate that for some individuals experiencing PAU in early midlife, this use may be the result of unique experiences/stressors in adulthood (Just‐Østergaard et al. 2018).

A major strength of this study was the prospective data collection following the same twin individuals across three decades of life. However, the results should be understood in the context of the following limitations. First, the time between the assessments spans several years. It is possible that individuals who were classified as never engaging in PAU may have had a period of problematic use that began and ended between assessments. Second, PAU was not consistently measured across all timepoints and samples, in part because the two‐stage sampling design of the FinnTwin12 cohort used different measurement approaches for PAU in the intensive sample compared to the epidemiological sample. We chose to harmonize across SSAGA, mmMAST, and AUDIT assessments to maximize sample size. However, we acknowledge that the use of different assessments at different timepoints and samples may have introduced error in our classification of problematic alcohol use. Third, we found that a majority of the sample (59%) experienced PAU at some point in their lives. A recent survey of drinking habits in Finland found that 28% of the population exceeded the lowest risk threshold of the AUDIT (Finnish Institute for Health and Welfare 2024). The high proportion of PAU in our sample may be the result of lifetime prospective assessment. While some longitudinal research has shown that experiencing at least one mental health disorder is more common than never experiencing a disorder over the lifetime (Caspi et al. 2020), we acknowledge that our findings may not extend to samples or populations with lower rates of PAU. Fourth, given the large sample sizes needed to detect interaction effects, we may have been underpowered to detect a significant moderating effect of sex on both the predictors and correlates of PAU trajectories. Finally, overall the sample is healthy in midlife which means the results presented may not generalize to populations experiencing more challenges in early midlife.

In summary, our findings demonstrate that PAU trajectories are associated with unique patterns of functioning and adaptation in early midlife. Results examining the genetic, behavioral, and environmental risk for PAU across the lifespan provide further support for multiple pathways to PAU. Encouragingly, our findings show that individuals who had remitted from PAU by early midlife closely resembled individuals who never experienced PAU, despite having elevated genetic and environmental risk. Additional research needs to be conducted to understand potential mechanisms for the development of PAU which onsets in early midlife.

## Funding

This study is supported by the National Institute on Alcohol Abuse and Alcoholism of the National Institutes of Health (Grant/Award Numbers: R01AA015416, K02AA018755 and K01AA024152) and the Research Council of Finland (Grant/Award Numbers: 100499, 205585, 118555, 141054, 265240, 263278, 264146, 308248, 352792, and 312073). Sarah Brislin is supported by the NIH Grant K23DA058808 from NIDA.

## Conflicts of Interest

Dr. Dick is a co‐founder of Thrive Genetics Inc., and a member of the advisory board of Seek Health Group Inc. She owns stock in both companies. The other authors declare no conflicts of interest.

## Supporting information


**Table S1:** Criteria for problem alcohol use across timepoints and samples.
**Table S2:** Additional information on the items that make up the behavioral and environmental risk index and endorsement rates by PAU group.
**Table S3:** Analytic sample descriptives stratified by sex.
**Table S4:** Estimates from the univariable models of behavioral and genetic predictors.

**Data S1:** Supplemental methods.

## Data Availability

Research data are not shared owing to Finnish data privacy laws.
