# Supplementary material for: Patterns, Predictors, and Correlates of Problematic Alcohol Use and Remission From Adolescence to Early Midlife
Source: Alcohol Clin Exp Res (Hoboken). 2026 Jun 30;50(7):e70365. doi: 10.1111/acer.70365 (PMC13316461; doi:10.1111/acer.70365)
Supplement: Supplementary file 2 — Data S1: Supplemental methods. [file ACER-50-0-s002.docx]

**Supplemental Methods**

**Early Midlife Correlates**

*Socioeconomic status indicators.* Twins reported their highest level of education achieved. Responses were recoded to compulsory education only, vocational secondary education, academic secondary school, and tertiary education (Cooke et al., 2025). Twins reported on their financial situation using an ordinal scale with 5 response options from “very good’ to “very poor”. Response options were coded such that higher scores represented a better financial situation.

*Relationship indicators.* Twins who reported living with a spouse or partner or reported currently being in a relationship were coded as partnered. Twins who reported being in a relationship were administered three subscales (relationship consensus, relationship satisfaction, and relationship cohesion) from the Revised Dyadic Adjustment Scale (RDAS; Busby et al., 1995). Relationship consensus (α = 0.69) measured the ability to achieve consensus in decision making in the relationship. Relationship satisfaction (α = 0.79) measured the extent to which they were satisfied in the relationship. Relationship cohesion (α = 0.73) measured the degree to which partners participate in activities together. All items were recoded (if necessary) such that higher scores represented more positive relationship outcomes. Items were then summed within each subscale. Twins reported whether they were a biological father/mother and whether there were non-biological children living in their household. Parenthood status was coded as a binary variable indicating whether the twin had biological children (regardless of residential status) or non-biological children living in the same household.

*Health related indicators.* Twins reported on their current health and current physical fitness using an ordinal scale with 5 response options from “very good” to “very poor” for each outcome (Silventoinen et al., 2007). Response options were coded such that higher scores represented better self-reported health or physical fitness. Twins reported the frequency of experiencing “headaches,” “low back pain,” “neck or shoulder pain”, “difficulty getting to sleep”, and “waking up during sleep” during the past 6 months using an ordinal scale with 5 response options from “less often or never” to “almost daily” (Mikkelsson et al., 1997). An endorsement of weekly or more frequent pain as “headaches,” “low back pain,” or “neck or shoulder pain” was coded as recurrent pain. An endorsement of weekly or more frequent difficulty getting to sleep or waking up during sleep was coded as recurrent sleep difficulties.

*Mental health related indicators.* Twins were administered the Satisfaction with Life Scale (Diener et al., 1985), a 5-item scale used to measure global cognitive judgments of one’s life satisfaction. Scores were summed to create a scale from 5 - 35, with a score of 20 being neutral (α = 0.89). Twins were administered the modified 8-item Center for Epidemiological Studies Depression Scale (CES-D; Radloff, 1977; Van de Velde et al., 2010), a self-report questionnaire designed to measure frequency of past-week depressive symptoms. Scores (0 to 3) were summed to create a range between 0 – 24, with higher scores indicating more severe and frequent depressive symptoms (α = 0.8).

References

Busby DM, Christensen C, Crane DR, Larson JH (1995) A Revision of the Dyadic Adjustment Scale for Use with Distressed and Nondistressed Couples: Construct Hierarchy and Multidimensional Scales. Journal of Marital and Family Therapy 21:289–308.

Cooke ME, Lumpe E, Stephenson M, Urjansson M, Aliev F, Palviainen T, Brislin SJ, Piirtola M, Rabinowitz J, Latvala A, Barr PB, Vuoksimaa E, Maes HHM, Viken R, Rose RJ, Kaprio J, Dick DM, Aaltonen S, Salvatore JE (2025) Alcohol use in Early Midlife: Findings from the Age 37 Follow-Up Assessment of the FinnTwin12 Cohort. Behav Genet 55:124–140.

Diener E, Emmons ,Robert A., Larsen ,Randy J., and Griffin S (1985) The Satisfaction With Life Scale. Journal of Personality Assessment 49:71–75.

Mikkelsson M, Salminen JJ, Kautiainen H (1997) Non-specific musculoskeletal pain in preadolescents. Prevalence and 1-year persistence. Pain 73:29–35.

Radloff L (1977) The CES-D Scale: A self-report depression scale for research in the general population. Appl Psychol Meas 1.

Silventoinen K, Posthuma D, Lahelma E, Rose RJ, Kaprio J (2007) Genetic and Environmental Factors Affecting Self-Rated Health from Age 16–25: A Longitudinal Study of Finnish Twins. Behav Genet 37:326–333.

Van de Velde S, Bracke P, Levecque K, Meuleman B (2010) Gender differences in depression in 25 European countries after eliminating measurement bias in the CES-D 8. Social Science Research 39:396–404.
